# Transparent and Colorless Luminescent Solar Concentrators Based on ZnO Quantum Dots for Building-Integrated Photovoltaics

**DOI:** 10.1021/acsomega.4c00772

**Published:** 2024-06-20

**Authors:** Manuel de J. Fimbres-Romero, Álvaro Flores-Pacheco, Mario E. Álvarez-Ramos, Rosendo Lopez-Delgado

**Affiliations:** † Departamento de Física, Universidad de Sonora, Hermosillo, Sonora 83000, México; ‡ Investigadores por México-CONAHCYT, CONAHCYT, Ciudad de México CP 03940, México

## Abstract

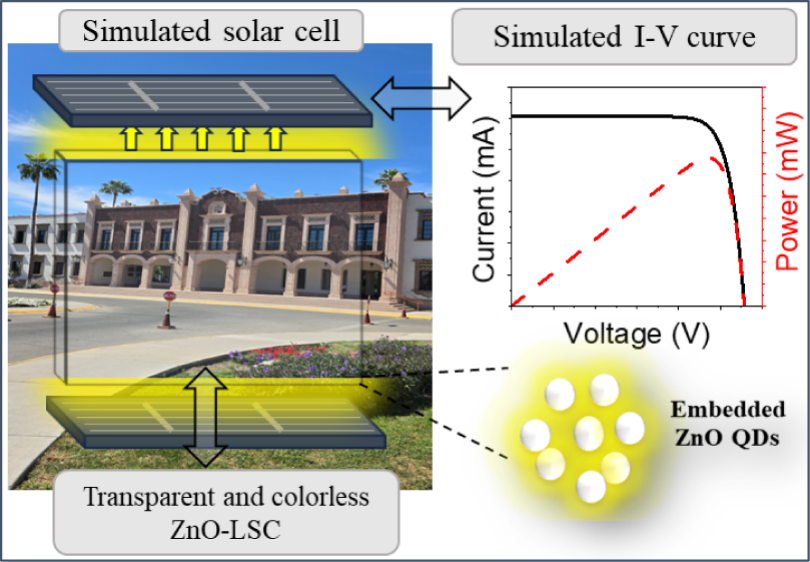

Scientific interest in luminescent solar concentrators (LSCs) has reemerged mainly due to the application of semiconductor quantum dots (QDs) as highly efficient luminophores. Recently, LSCs have become attractive proposals for Building-Integrated photovoltaics (BIPV) since they could help conventional photovoltaics to improve sunlight harvesting and reduce production costs. However, most of the modern LSCs rely on heavy-metal QDs which are highly toxic and may cause environmental concerns. Additionally, their absorption spectra give them a characteristic color limiting their potential application in BIPV. Herein, we fabricated transparent and colorless LSCs by embedding nontoxic and cost-effective zinc oxide quantum dots (ZnO QDs) in a PMMA polymer matrix (ZnO-LSC), preserving the QD optical properties and PMMA transparency. The synthesized colloidal ZnO QDs have an average size of 5.5 nm, a hexagonal wurtzite crystalline structure, a broad yellow photoluminescent signal under ultraviolet excitation, and are highly visibly transparent at the employed concentrations (>95% in wavelengths above 400 nm). The optical characterization of the fabricated ZnO-LSCs showed a good visible transparency of 80.3% average visible transmission (AVT), with an LSC concentration factor (*C*) of 1.02. An optimal device (ZnO-LSC-O) could reach a *C* value of 2.66 with the combination of optical properties of colloidal ZnO QDs and PMMA. Finally, simulations of the performance of silicon solar cells coupled to the fabricated and optimal LSCs under standard AM 1.5G illumination were performed employing the software COMSOL Multiphysics. The fabricated ZnO-LSC achieved a simulated maximum power conversion efficiency (PCE) of 3.80%, while the optimal ZnO-LSC-O reached 5.45%. Also, the ZnO-LSC generated a maximum power of 15.02 mW and the ZnO-LSC-O generated 40.33 mW, employing the same active area as the simulated solar cell directly illuminated, which generated 14.39 mW. These results indicate that the ZnO QD-based LSCs may be useful as transparent photovoltaic windows for BIPV applications.

## 1 Introduction

Solar irradiation is an abundant and natural source of energy with high potential for sustainable power generation^1,2^ and one of the most promising candidates to supplant oil due to the high solar irradiation reaching the earth’s surface (140000 TWh).^3^ Therefore, improvements in solar energy harvesting, and principally photovoltaic technology, are active developing topics aimed at increasing conversion efficiencies and reducing costs.^4,5^ Besides conventional photovoltaic systems, some devices could help to harvest sunlight more efficiently by using inexpensive materials such as luminescent solar concentrators (LSCs). These devices can absorb sunlight and emit light by photoluminescence, which is guided by total internal reflection to the device’s edges, where coupled solar cells convert sunlight into electricity.^6,7^ LSCs are normally made with a lumiphore, which is the material responsible for absorbing and emitting sunlight, and a transparent polymer matrix responsible for waveguiding the luminescence light to the edges. LSCs have become very attractive devices for Building-Integrated photovoltaics (BIPV), since they can be used in the form of transparent photovoltaic windows or envelope components.^8−10^ In recent years, LSC researchers have found other areas of application in “agrivoltaics”, which is the use of large areas of land for photovoltaic and agricultural purposes. In this type of application, LSCs harvest a portion of the sunlight in greenhouses, helping to improve the plant growth since LSCs can absorb specific regions of sunlight and allow the rest to be transmitted to the plants.^11,12^ LSCs were initially proposed in the late 1970s^13^ but were inefficient due to the intrinsic properties of the lumiphores employed, such as organic dyes that present very high reabsorption losses and rare earth ions with very narrow absorption spectra.^14,15^ In recent years, semiconductor nanoparticles also called quantum dots have been developed as lumiphores for LSC technology due to its attractive properties, such as high quantum yield, tunable absorption, and photoluminescence spectra and large Stokes shift, helping LSCs to reemerge as an active topic.^16,17^ LSCs based on QDs have been reported by embedding diverse types of QDs in polymers, such as, carbon dots in PMMA matrix, and CuGaAlS/ZnS core/shell quantum dots in a polylauryl-methacrylate;^18−20^ however, besides the toxicity concerns, most of them present absorption in visible light region, presenting a characteristic color, decreasing their transparency and limiting their use in BIPV technologies as photovoltaic windows. Among the various lumiphores investigated for LSC applications, zinc oxide quantum dots (ZnO QDs) have appeared as promising candidates due to their unique optical properties, such as tunable photoemission, high photoluminescence quantum yield, and excellent photostability.^21,22^ Despite the limited absorption spectral region of ZnO due to its wide bandgap (3.3 eV), ZnO QDs present great absorption of photons in the high energy range of the solar spectrum, where a considerable amount of solar energy is available. In fact, below 375 nm, the AM1.5G solar irradiance accounts for more than 28 W/m^2^ of power density that could be used to produce photovoltaic energy. Also, such a large bandgap allows the absorption of ultraviolet light (a region where silicon solar cells perform poorly), while letting the visible light to be transmitted, enabling the fabrication of attractive transparent and colorless LSC devices that could be employed as photovoltaic windows in BIPV technologies. Other advantages of ZnO QDs as lumiphore for LSCs include: their characteristic photoluminescence signal, located in the visible region, which is better suitable for the absorption of conventional silicon solar cells; their ecofriendly synthesis process;^23−25^ and the ease to obtain nanoparticles with controlled size, shape, and surface properties, giving the opportunity to match the specific requirements of different LSC devices.^26−29^ In this study, we synthesized luminescent ZnO QDs and fabricated colorless and highly transparent LSCs based on ZnO QDs as lumiphore and poly(methyl methacrylate) (PMMA) as transparent matrix (ZnO-LSC). Also, a combination of optical properties was proposed to obtain an optimal ZnO-LSC-O device. Finally, the COMSOL Multiphysics semiconductor module was used to simulate the photovoltaic performance of silicon solar cells coupled to the fabricated and optimal LSCs under a standard AM 1.5G illumination.

## 2 Experimental Section

### 2.1 Materials and Characterization Methods

Zinc acetate dihydrate (Zn (CH_3_COO)_2_·2H_2_O, > 98%), sodium hydroxide (NaOH, > 98%), benzoyl peroxide (BPO, >98%), and methyl methacrylate (MMA, > 99%) were purchased from Sigma-Aldrich. Hexane (C_6_H_14_, > 99%) and ethanol anhydrous (CH_3_CH_2_OH > 99%) were obtained from Baker ACS. All precursors were used as purchased without any further treatment.

The structural characterization and size determination of ZnO QDs were performed by X-ray diffraction (XRD) employing a Cu-kα source (λ = 1.5406 Å) at a scan rate of 0.1°/s from 20° to 80° of 2θ and a dynamic light scattering (DLS) method employing a Malvern Zetasizer Nano-ZS, respectively. Fourier transform infrared (FT-IR) and Raman vibrational spectroscopy were measured with a PerkinElmer Spectrum Two FT-IR in attenuated total reflectance (ATR) and a Horiba LabRam HR spectrometer, respectively. The chemical composition was analyzed by X-ray photoelectron spectroscopy (XPS) employing a PerkinElmer PHI 5100 with an Mg anode generating a Kα radiation of 1253.6 eV. Transmittance, absorption, and reflectance spectra were measured with a PerkinElmer Lambda 365 UV–vis spectrometer. Photoluminescence properties were measured with a Horiba iHR-320 spectrofluorometer and a Hamamatsu R928 multialkali photomultiplier as detector, where a 450 W xenon arc lamp coupled to a Triax 320 monochromator and a He–Cd laser (325 nm) were used as excitation sources.

### 2.2 Synthesis of ZnO Quantum Dots

Zinc oxide quantum dots were synthesized by the sol–gel method described elsewhere with few modifications.^30^ To this end, a 40 mM solution of zinc acetate dihydrate (Zn (CH_3_COO)_2_·2H_2_O) was made by dissolving 0.220 g of zinc acetate in 25 mL of anhydrous ethanol and stirred for 30 min. A 40 mM solution of sodium hydroxide (NaOH) was prepared separately by dissolving 0.040 g of NaOH in 25 mL of ethanol anhydrous. Afterward, the 25 mL solution of NaOH was mixed with the zinc acetate solution and the synthesis was carried out by magnetic stirring at room temperature for 2 h. A visible transparent, colloidal solution of ZnO quantum dots was obtained. The ZnO QDs were purified by centrifugation for 15 min at 10000 rpm at a temperature of 0 °C employing hexane a nonpolar solvent (volume ratio of 2:1). The supernatant was removed and the ZnO QD precipitate was redispersed in pure ethanol.

### 2.3 PMMA and ZnO-LSC Fabrication

**Scheme 1:**
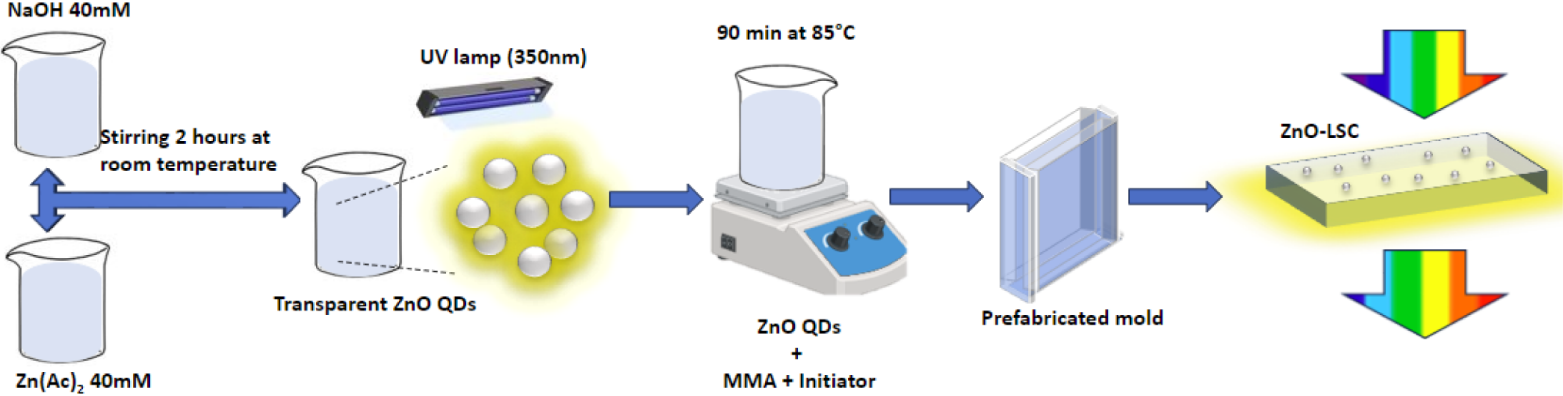
Experimental Methodology for the Fabrication of Colorless-Transparent LSC Based on ZnO QDs

Reference PMMA slabs were obtained following the Bagherzadeh method with some modifications.^31^ Here, 15 mL of methyl methacrylate (MMA) was poured into a beaker and 0.8 wt % of benzoyl peroxide (BPO) was added as a polymerization initiator. The solution was then heated at 85 °C for 90 min, and the solution began to obtain a high viscosity, indicating the formation of the prepolymer. At this point, the solution was poured into a preassembled mold and left to polymerize at 60 °C for 24 h. Finally, the mold was disassembled to obtain the PMMA slab. The mold was designed to create 3.33 cm wide and 3 mm thick slabs with variable lengths, which is the standard thickness of building windows.^32^ The coupling of ZnO quantum dots with the PMMA matrix to create the ZnO-LSC was carried out with a similar procedure by adding 2 mL of purified ZnO QDs after 60 min of heating the MMA with the initiator (before prepolymerization). This last step was performed to obtain a homogeneous distribution of ZnO QDs within the polymeric matrix. After the formation of prepolymer, the ZnO QDs and PMMA mix was poured into the preassembled mold and left to polymerize at 60 °C for 24 h. The mold was then disassembled to obtain the ZnO-LSC device. In addition to the fabricated PMMA slabs and ZnO-LSCs, an optimal ZnO-LSC-O was proposed by the combination of optical properties of pure PMMA and colloidal ZnO QDs. The overall experimental methodology is depicted in Scheme 1.

### 2.4 Optical Model and Photovoltaic Simulation Details

To evaluate LSC performance, key parameters are optical efficiency (*η*_opt_) defined as the ratio of photon flux that reaches the edges of the LSC (Φ_2_) to the total incident photon flux (Φ_1_) and the concentration factor (*C*) defined as the ratio of photon flux density reaching the edges of the LSC (ϕ_2_= Φ_2_/*A*_PV_) to the photon flux density incident on LSC surface (ϕ_1_= Φ_1_/*A*_LSC_), were *A*_PV_ is the area of the LSC edge (or the coupled photovoltaic area) and *A*_LSC_ is the area of the LSC exposed surface. As proposed by Klimov et al. and further employed by Flores-Pacheco et al.,^33,34^ these two parameters could be calculated with the LSC optical properties by the equations:
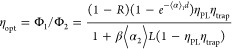

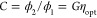
where *R* is the LSC experimental reflectance, ⟨α_1_⟩ is the irradiance-dependent absorption coefficient, ⟨α_2_⟩ is the photoluminescent dependent absorption coefficient, *η*_PL_ is the quantum yield of the lumiphore, *η*_trap_ is the refractive index-dependent total internal reflection light trapping efficiency, β accounts for the scattering losses of the waveguide, and *G* = *A*_LSC_/*A*_PV_ is the geometric factor. Considering Klimov’s geometry, with two perfectly reflective mirrors, *G* = *L*/2*d*, where *L* and *d* are the LSC length and thickness, respectively.

To simulate the performance of the ZnO-LSC and ZnO-LSC-O coupled to photovoltaic devices, a reference silicon solar cell was simulated by employing the semiconductor module of the software COMSOL Multiphysics. The 1D solar cell model proposed by Flores-Pacheco was used with a few modifications.^34^ Briefly, the simulated solar cell started from a 200 μm thick, n-type silicon base (donor concentration *N*_D_ = 3 × 10^14^ cm^–3^), then, a 50 nm deep p-type region was created on the front surface with an acceptor concentration of *N*_A_ = 3 × 10^20^ cm^–3^ to simulate the p-n homojunction. The simulated reference silicon solar cell was proposed with an active area of 1.0 cm^2^ (3.33 cm wide and 3 mm thick) to fit with the fabricated LSC slab dimensions. The *I*–*V* characteristics of the simulated reference silicon solar cell were obtained with a forward-biased voltage (0–0.65 V) under a standard AM1.5G solar irradiation spectrum (100 mW/cm^2^).

## 3 Result and Discussion

### 3.1 Structural Characterization

**Figure 1:**
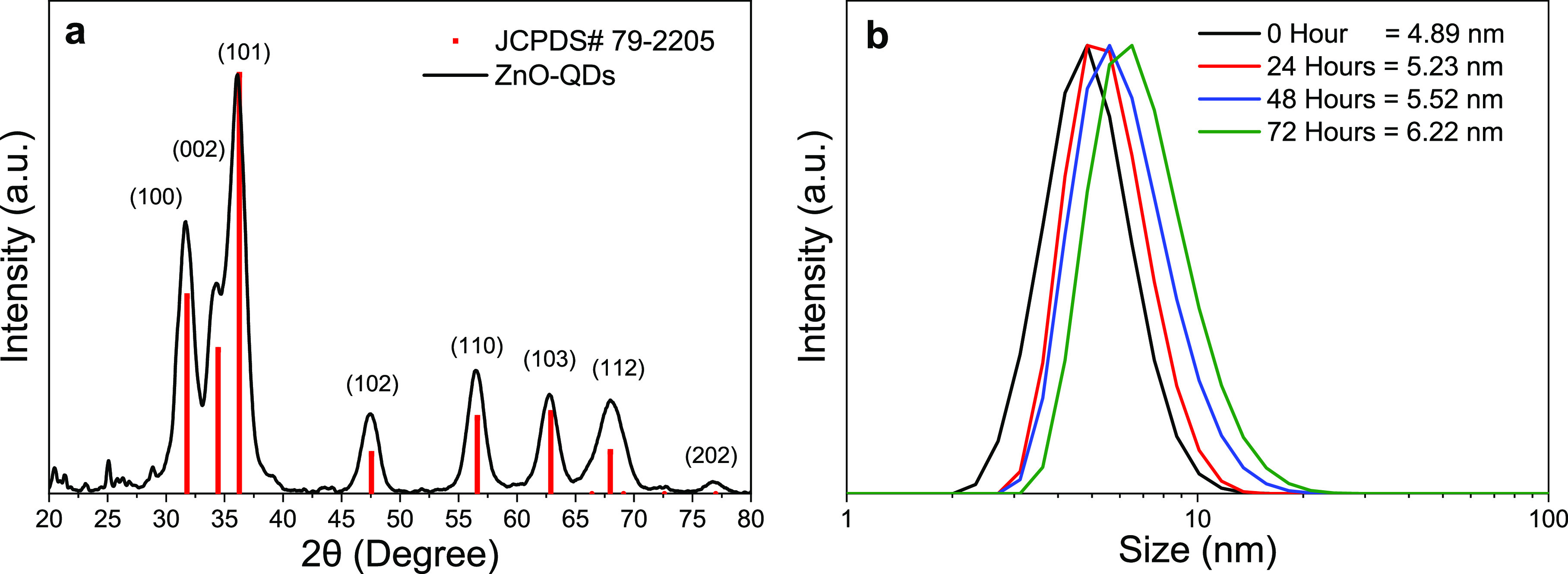
(a) X-ray diffraction pattern and (b) DLS size measurements of ZnO QDs.

X-ray diffraction (XRD) characterization was performed to analyze the crystalline structure of the ZnO quantum dots (Figure 1a). According to the JCPDS#79-2205 card, the characteristic peaks at 31.6°, 34.6°, 36.2°, 47.4°, 56.5°, 62.8°, 66.0°, 76.8° correspond to the crystalline planes (100), (002), (101), (102), (103), (112), and (202), respectively, indicating the widely reported ZnO hexagonal wurtzite structure.^35,36^ It is well-known that the width of the X-ray diffraction peaks is size dependent; the larger crystals have narrower peaks while broad peaks are normally observed for nanostructured materials. According to this, crystallite size was calculated analyzing the XRD patterns and the Scherrer’s equation:^37^
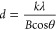
where *d* is the crystallite size, *k* is a shape-dependent constant (equal to 0.9 for spheric nanostructures), λ is the wavelength of the Cu Kα radiation (0.154 nm), *B* is the full width at half-maximum intensity of the peak (fwhm), and θ is the Bragg’s diffraction angle. Applying a deconvolution to the diffraction peaks to obtain positions θ and fwhm *B* and analyzing the collection of peaks, the calculated crystallite size of the ZnO quantum dots is 5.3 ± 0.15 nm.

The size distribution of ZnO QDs was also obtained by dynamic light scattering (DLS) as shown in Figure 1b. To analyze the size stability of the QDs without the cleaning process, the DLS measurement was performed immediately at the end of the reaction and every 24 h. The QDs presented an initial average diameter of 4.89 ± 1.3 nm (0 h), and as the colloidal solution aged, the sizes increased to 5.23 ± 1.4 (24 h), 5.52 ± 1.2 (48 h), and 6.22 ± 2.07 nm (72 h). After 72 h, the highly transparent QD solution became turbid, indicating the agglomeration of quantum dots and losing their attractive properties for transparent LSC applications. The gradual size increase and later agglomeration of the nonpurified ZnO QDs is thought to be produced by a residual reaction of the unreacted subproducts, since the average QD size did not change for the purified ZnO QDs, maintaining an average diameter of 5.5 ± 1.6 nm, which agrees with XRD results and previous investigations.^28,38^

**Figure 2:**
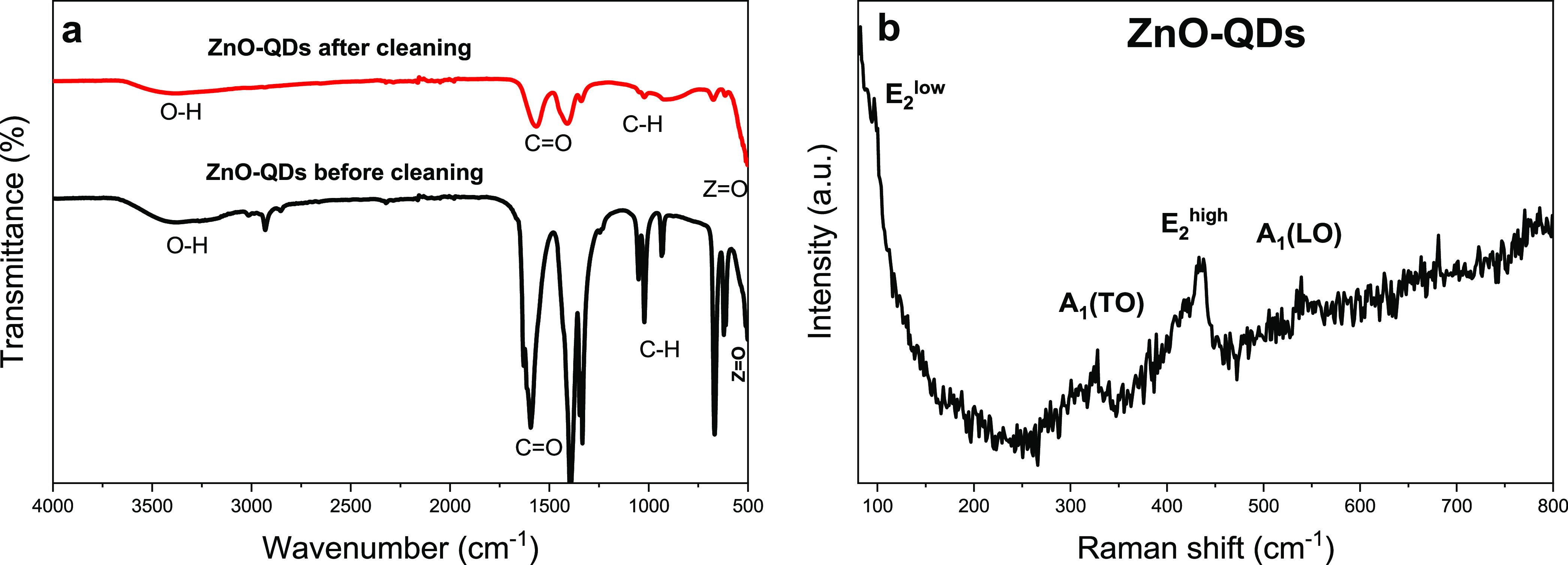
(a) FT-IR spectra before and after the precipitation process and (b) Raman spectra of the ZnO QDs.

FT-IR and Raman vibrational spectroscopies were conducted to further analyze the synthesized ZnO QDs. FT-IR spectrum in Figure 2a shows the functional groups present in ZnO QDs. The stretching mode of the hydroxyl group (O–H) was found at 3500 cm^–1^, the signals present in the 1400–1600 cm^–1^ range correspond to the C=O asymmetrical and symmetrical stretching modes and the signals present in 1050–900 cm^–1^ correspond to stretching mode of C–H. All these signals are functional groups from water and acetate, present on the surface of ZnO QDs.^39,40^ The peak near 500 cm^–1^ is attributed to the Zn–O bond.^41,42^ FT-IR measurements were performed before and after the cleaning process, and it could be observed that after cleaning, the peaks attributed to unreacted subproducts decreased while the peak near 500 cm^–1^ of ZnO was enhanced, which agrees with the DLS findings. Raman spectrum in Figure 2b was used as a complementary technique to identify ZnO QD characteristic vibrational modes. The two signals appearing in 100 and 440 cm^–1^ are related to the E_2_ low and high modes, respectively, and the signals at 338 and 558 cm^–1^ correspond to A_1_ (TO) and A_1_ (LO), respectively. All these signals are typical to the hexagonal phase of ZnO.^43^

**Figure 3:**
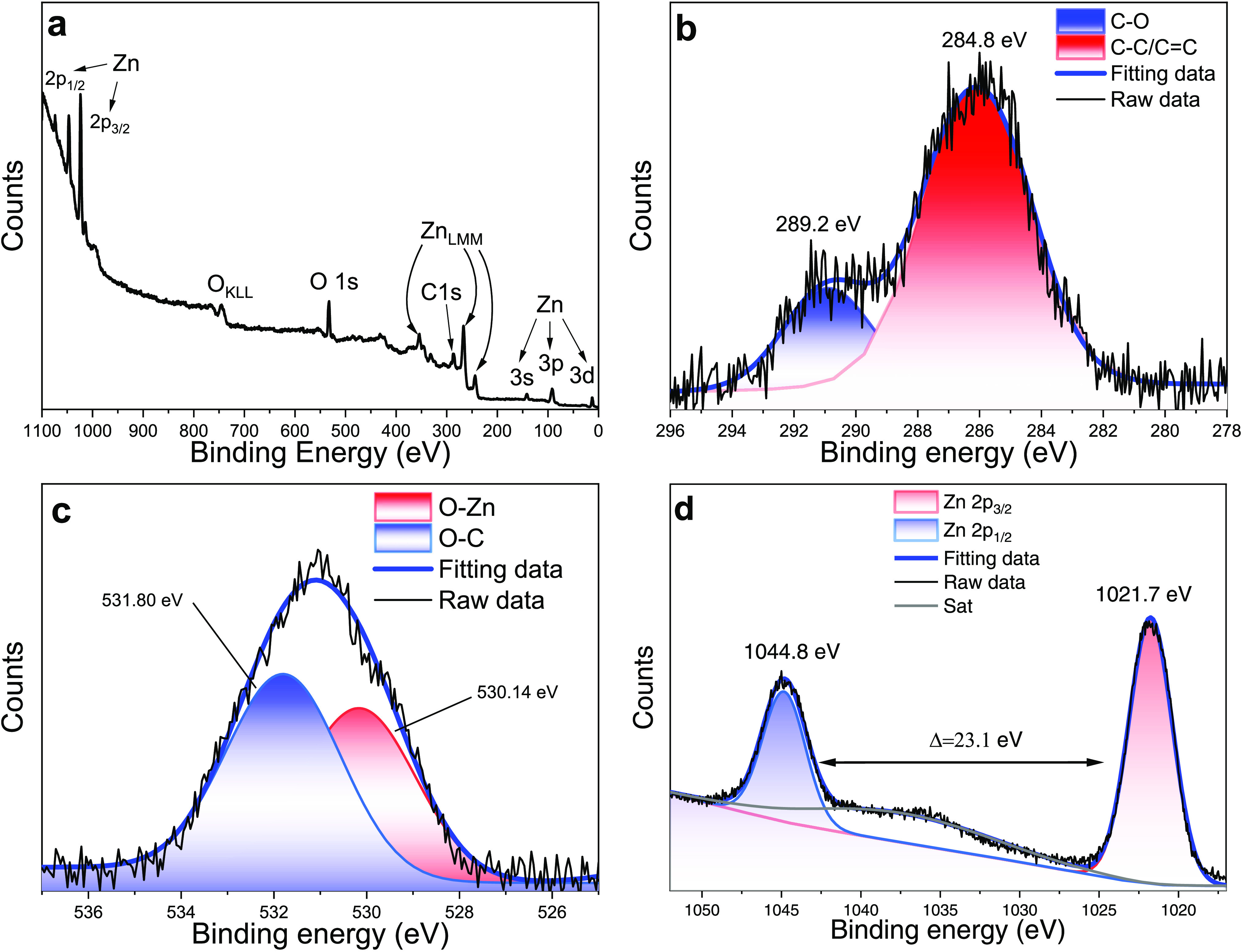
XPS spectra of ZnO quantum dots: (a) survey spectra, high resolution of (b) C 1s, (c) O 1s, and (d) Zn 2p signals.

The ZnO quantum dots were examined by X-ray photoelectron spectroscopy (XPS) to further analyze their chemical composition. Figure 3a shows the survey spectra of ZnO QDs where the main signals of Zn, O, Na, and C were found. High-resolution spectra of the C 1s, O 1s and Zn 2p are shown in Figure 3b–d. The charge shift was corrected using the C 1s peak of graphitic carbon (binding energy = 284.8 eV) and the signal centered at 289.2 eV of the C–O bond was associated with the acetate groups bounded on the ZnO QDs surface.^44^Figure 3c shows the O 1s signal deconvoluted in two components centered at 530.14 and 531.80 eV, where the low binding energy peak is attributed to O^2–^ ions on the wurtzite structure from Zn–O bonding^45,46^ and the high binding energy peak could be attributed to O^2–^ ions in defect regions and to hydroxyl groups (O–H),^47^ which is in agreement with the FTIR analysis. Figure 3d shows the Zn 2p spectra with two symmetric peaks centered at 1021.7 and 1044.8 eV, corresponding to the spin–orbit coupling of the Zn 2p_3/2_ and Zn 2p_1/2_ levels, respectively.^26^ The binding energy separation of 23.1 eV between the peaks confirms the state of Zn ions on the wurtzite structure from the ZnO QDs.

### 3.2 Optical Characterization

#### 3.2.1 Colloidal ZnO QDs

**Figure 4:**
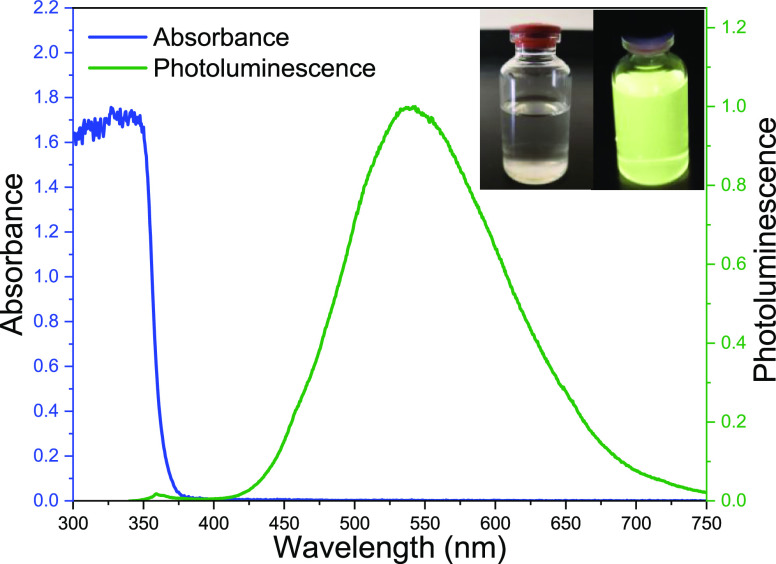
Absorbance and photoluminescent spectrum of colloidal ZnO QDs under excitation of 350 nm. Inset: ZnO QDs under white and UV light.

Some of the desired properties of lumiphores to be used in transparent LSC technology are low absorption in the visible range of the electromagnetic spectrum, a luminescent spectrum in a wavelength range compatible with the attached solar cell, and a minimum overlap between their absorption and luminescent spectra. As shown in Figure 4, the colloidal ZnO QDs show the characteristic strong absorption of photons below 375 nm, in the ultraviolet range, while a practically negligible absorption in the visible range, a property that makes the synthesized material colorless and highly transparent, as corroborated in the inset picture. Figure 4 also shows the photoluminescence spectrum of ZnO QDs in solutions under an excitation of 325 nm. Here, two signals are identified: the characteristics excitonic peak at around 360 nm, and the broad luminescent band located between 425 and 750 with a maximum at around 540 nm attributed to the collection of defects, as reported elsewhere.^40,48^ The inset picture displays the colloidal ZnO QDs under visible and ultraviolet excitation, showing high transparency and yellow luminescence produced by the defect band. The absorption and luminescence spectra of the synthesized ZnO QDs fulfill some of the most important properties to be considered as great candidates for luminophores in transparent LSC technology.

#### 3.2.2 PMMA and ZnO-LSC Reflectance, Transmittance, and Luminescence

**Figure 5:**
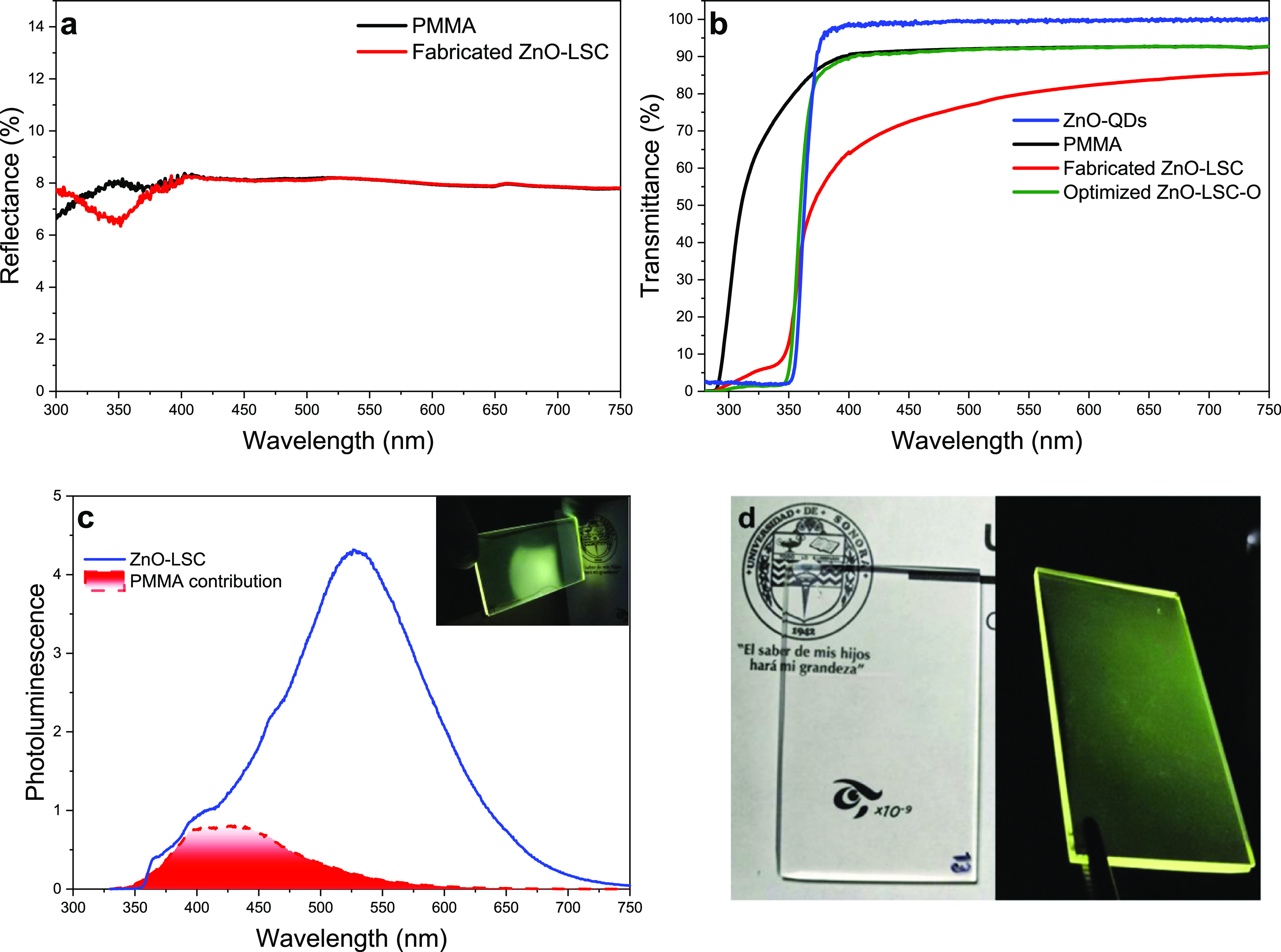
(a) Reflectance of PMMA slabs and fabricated ZnO-LSC, (b) transmittance of PMMA, ZnO QDs, ZnO-LSC, and ZnO-LSC-O, (c) photoluminescence of the fabricated ZnO-LSC under 350 nm excitation, (d) a picture of the device under white and UV light and its concentration properties.

Figure 5 shows the optical properties of PMMA and ZnO-LSC. The reflectance spectrum of both PMMA and ZnO-LSC has a practically constant value of 8% in the range of 400–800 nm (Figure 5a). The slight decrease in reflectance around 350 nm in the ZnO-LSC could be attributed to the absorption of ZnO QDs located near the device’s surface. The high transmittance of PMMA slabs (black line in Figure 5b) demonstrates a great degree of transparency in the visible spectrum (>90%), making it an appropriate base material for transparent LSCs. The fabricated ZnO-LSC presented a slight transmittance decrease in the visible region due to a possible agglomeration of ZnO QDs during the polymerization process, slightly reducing the transparency compared to pure PMMA. It is known that PMMA has excellent UV shielding qualities in wavelengths below 300^49^ but with the addition of ZnO QDs, the ZnO-LSC produced an improvement in UV shielding up to 350 nm, enhancing its potential as photovoltaic windows. In the optimal ZnO-LSC-O, the transmittance of the PMMA between 400 and 800 nm remained unchanged (which is achievable since the ZnO QDs do not absorb in this range), while the transmittance edge was shifted from 300 to 350 nm due to the ZnO QD absorption.

Figure 5c shows the photoluminescence spectrum of the fabricated ZnO-LSC, measured at the edge of the device. It was found that the luminescence of the ZnO QDs remained nearly unchanged, with a maximum at 540 nm corresponding to the characteristic defect band between 400 and 750 nm; however, since PMMA also exhibits a small blue photoluminescence when excited with 350 nm (red shading), the LSC luminescence presented a small signal between 400 and 450 nm. Figure 5d presents a picture of the fabricated ZnO-LSC under white light, showing the colorless and high transparency and the yellow luminescence under UV excitation of 350 nm attributed to the embedded ZnO QDs in the PMMA matrix.

#### 3.2.3 Colloidal ZnO QDs and ZnO-LSC Time-Resolved Photoluminescence

**Figure 6:**
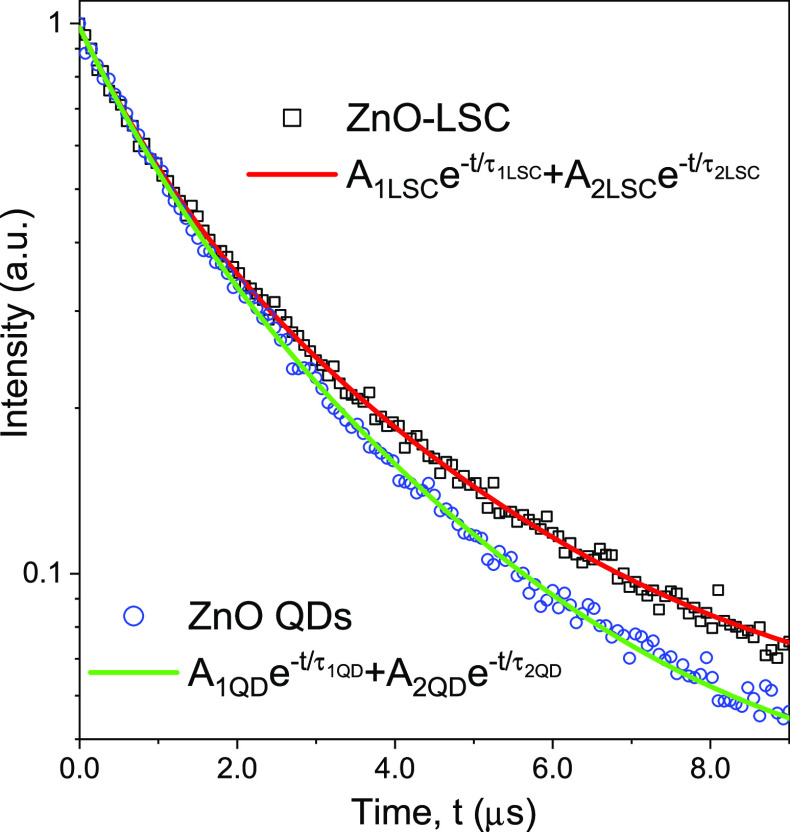
Photoluminescent decay curves of colloidal ZnO QDs and ZnO-LSC devices.

Photoluminescent lifetime measurements were performed on both the colloidal ZnO QD solution and the ZnO-LSC device to analyze if there was a modification in the luminescent properties of the QDs after the transfer to the LSC. The time-resolved luminescence was monitored at the wavelength of maximum intensity (540 nm). As shown in Figure 6, different decay times were found for the colloidal ZnO QDs and ZnO-LSC. In both cases, the lifetimes were fitted to a biexponential decay function , where *I*(*t*) represents the luminescent intensity; τ_1_ and τ_2_ are the fast and slow decay times, respectively; and *A*_1_ and *A*_2_ are the amplitudes of the fast and slow decay profiles, respectively. It has been reported that the fastest decay component depends on the size of the ZnO nanoparticle.^50^ Here, the τ_1_ values for the colloidal QDs and LSC were 846 and 839 ns, respectively, indicating that the inclusion of the QDs in the LSC does not produce a significant change in their size. The lifetimes of the slowest decay component τ_2_ were 2506 and 2811 ns for the colloidal QDs and LSC, respectively, indicating longer-lived excited states in the LSC due to a possible reduced QD interaction, preventing the nonradiative recombination.^51^ Also, the average photoluminescent lifetime was calculated as .^52^ The average lifetimes also increased from 2208 ns for the colloidal QDs to 2450 ns for the LSC.

#### 3.2.4 PMMA and ZnO-LSC Visible Transparency

According to Yang et al.,^53^ the transparency of an LSC can be evaluated with the average visible transmission (AVT), a parameter that describes the effective transmission of visible light from a specified illumination source. Considering the solar radiation as illumination source, the AVT is calculated by the equation:
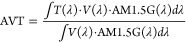


where *T*(λ) is the transmission spectrum of the LSC, *V*(λ) is the photopic response, and AM1.5G(λ) is the solar standard photon flux. The AVT was calculated for both pure PMMA and ZnO-LSC, obtaining values of 92.3% and 80.3%, respectively. Even though there is a decrease in the AVT of the LSC, a value of 80.3% is still considered as a good transparent window.^54^ Besides the fabricated devices, the AVT was also calculated for ZnO-LSC-O, obtaining a value of 91.1%.

### 3.3 Optical Efficiency and Concentration Factor

**Figure 7:**
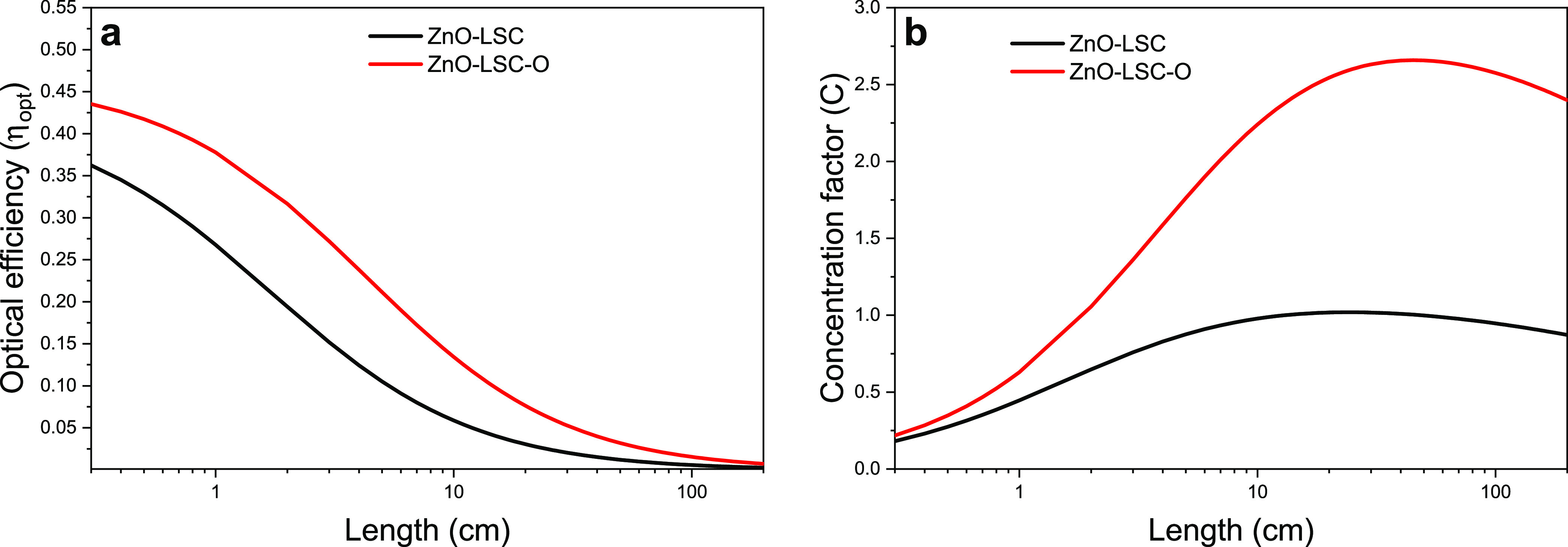
(a) Optical efficiency and (b) concentration factor for LSC.

The optical efficiency *η*_opt_ and concentration factor *C* values were calculated using the experimental data of reflectance *R* = 0.08; the absorption coefficients ⟨α_1_⟩ of 7.09 and 11.03 for the fabricated and optimal LSC, respectively, and ⟨α_2_⟩ of 0.86 and 0.34 for fabricated and optimal LSC, respectively; a light trapping efficiency of *η*_trap_ = 0.75, which correspond to a PMMA slab with refractive index of 1.5;^33,34^ and a photoluminescent quantum yield of *η*_PL_*=* 0.70 was employed since this parameter was obtained by a similar synthesis route, with the same precursors (zinc acetate and a metallic hydroxide) and the same chemical reaction.^55^Figure 7 shows the values of *η*_opt_ and *C* calculated for the fabricated ZnO-LSC and optimal ZnO-LSC-O for different lengths. As expected, *η*_opt_ decreases as the length increases since a larger photon path leads to a higher probability of losses through the different process before reaching the edges. On the other hand, Figure 7b shows that the concentration factor could be increased due to the geometric factor up to a maximum point; then, the losses and reabsorption start to become greater than the geometric contribution and *C* begins to decrease. The calculated maximum *C* values were 1.019 for the fabricated ZnO-LSC and 2.66 for optimal ZnO-LSC-O for LSC lengths of 25 and 50 cm, respectively. It is important to mention that in both cases *C***>** 1, implying that both devices can effectively concentrate the incident photon flux density to the edges.

### 3.4 Photovoltaic Simulation of LSC-PV Devices

The current voltage (*I*–*V*) characteristics and photovoltaic performance of the simulated reference silicon solar cell under standard AM1.5G solar irradiation spectrum (100 mW/cm^2^) is summarized in Table 1. The simulated reference performs as a commercially available silicon solar cell, generating 14.39 mW with an active area of 1.0 cm^2^.

**Table 1:** *I*–*V* Characteristics of the Simulated Reference Silicon Solar Cell Under Direct AM1.5G Solar Irradiation

PV area (cm^2^)	*P*_in_ (mW)	*P*_out_ (mW)	*V*_oc_ (V)	*I*_sc_ (mA)	FF (%)	PCE (%**)**
1.0	100	14.39	0.590	32.30	75.5	14.39

**Figure 8:**
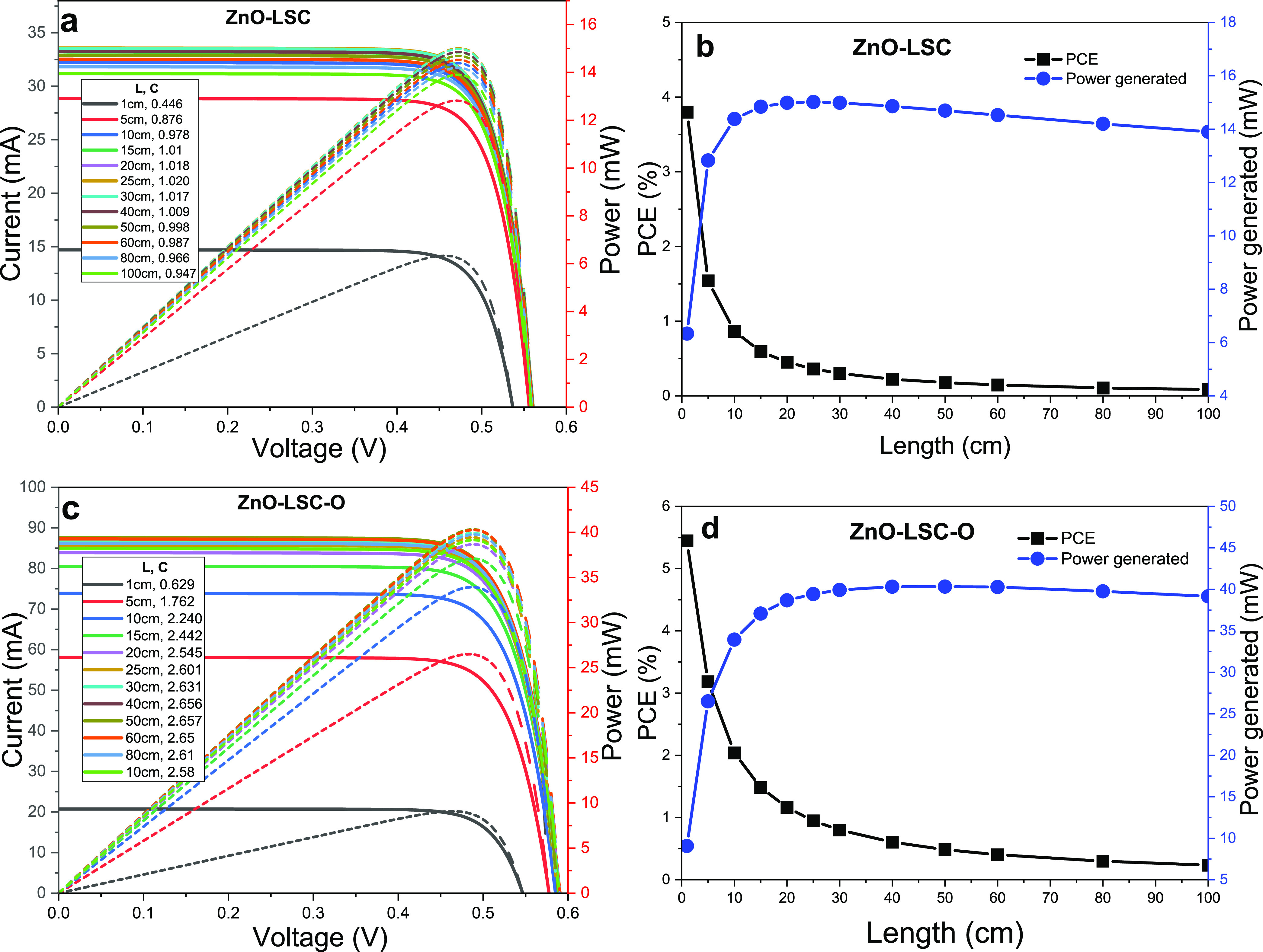
(a, c) *I*–*V* characteristics and (b, d) PCE and power generation as a function of length of the fabricated ZnO-LSC and optimal ZnO-LSC-O.

Figure 8 shows the simulated *I*–*V* characteristics, power conversion efficiency (PCE) (defined as PCE = *P*_out_/*P*_in_, where *P*_in_ = *A*_LSC_ × 100 mW/cm^2^), and the power generation (*P*_out_) curves of the fabricated ZnO-LSC and optimized ZnO-LSC-O systems for different LSC lengths. As shown in Figure 8b, the PCE decreases from 3.80% to 0.08% as the ZnO-LSC length increases from 1 to 100 cm, while the *P*_out_ exhibits a tendency to increase from 6.33 mW for a 1 cm device up to a maximum value of 15.02 mW for a 25 cm device, then, the *P*_out_ decreases to a value of 13.90 mW for a device of 100 cm.

Figure 8c,d shows the photovoltaic performance of the optimal ZnO-LSC-O-PV systems, where enhanced performances but with similar trends as ZnO-LSC-PV were found. Here, the PCE values decreased from 5.45% to 0.24% as the ZnO-LSC-O length increased from 1 to 100 cm, while the *P*_out_ increased from 9.08 mW for a 1 cm device up to a maximum of 40.33 mW for a 50 cm device, then, the *P*_out_ decreased to 39.16 mW for a device of 100 cm.

Even though the PCE values of both the fabricated ZnO-LSC-PV and optimized ZnO-LSC-O-PV systems (up to 3.80% and 5.45%, respectively) are lower than the 14.39% found for the solar cell under direct illumination, ZnO-LSC-PV can generate a maximum power of 15.02 mW and the ZnO-LSC-O-PV up to 40.33 mW employing the same photovoltaic active area (1.0 cm^2^), demonstrating that these ZnO-based LSC devices are capable of concentrating solar radiation and generating more power than the 14.39 mW generated by the same solar cell directly exposed. It is important to notice that the fabricated ZnO-LSC devices (with lengths between 15 and 60 cm) and the optimal ZnO-LSC-O devices (with L > 10 cm) can generate more power than the same solar cell directly exposed, while functioning as highly transparent photovoltaic windows according to their AVT values. Tables 2 and 3 show the simulated *I*–*V* parameters of the fabricated ZnO-LSC and optimal ZnO-LSC-O devices, respectively.

**Table 2:** Optical Performance and *I*–*V* Parameters of the Fabricated ZnO-LSC-PV Systems

length (cm)	*η*_opt_	*C*	*P*_in_(mW)	*P*_out_(mW)	*V*_oc_ (V)	**I**_**sc**_**(mA)**	FF(%)	PCE(%)
1	0.268	0.446	333.3	6.33	0.535	14.70	80.5	3.80
5	0.105	0.876	1665	12.82	0.555	28.86	80.1	1.54
10	0.059	0.978	3330	14.38	0.560	32.22	79.7	0.86
15	0.040	1.008	5000	14.84	0.561	33.21	79.7	0.59
20	0.031	1.017	6660	14.99	0.562	33.53	79.6	0.45
25	0.024	1.019	8325	15.02	0.564	33.58	79.3	0.36
30	0.020	1.017	9990	14.99	0.564	33.52	79.3	0.30
40	0.015	1.009	13320	14.86	0.563	33.24	79.4	0.22
50	0.012	0.998	16650	14.69	0.563	32.89	79.3	0.18
60	0.010	0.987	19980	14.52	0.559	32.53	79.9	0.15
80	0.007	0.966	26640	14.20	0.558	31.83	79.9	0.11
100	0.006	0.947	33300	13.90	0.558	31.19	79.9	0.08

**Table 3:** Optical Performance and *I*–*V* Parameters of the Optimized ZnO-LSC-O-PV Systems

length (cm)	*η*_opt_	*C*	*P*_in_(mW)	*P*_out_ (mW)	*V*_oc_ (V)	**I**_**sc**_**(mA)**	FF (%)	PCE(%)
1	0.378	0.63	333.3	9.08	0.545	20.74	80.3	5.45
5	0.211	1.76	1665	26.50	0.578	58.09	78.9	3.18
10	0.134	2.24	3330	33.94	0.582	73.86	79.0	2.04
15	0.098	2.44	5000	37.09	0.586	80.53	78.6	1.48
20	0.076	2.54	6660	38.68	0.586	83.90	78.7	1.16
25	0.062	2.60	8325	39.44	0.589	85.73	78.1	0.95
30	0.053	2.63	9990	39.92	0.588	86.76	78.3	0.80
40	0.040	2.66	13320	40.31	0.587	87.58	78.4	0.61
50	0.032	2.66	16650	40.33	0.587	87.61	78.4	0.48
60	0.026	2.65	19980	40.28	0.59	87.29	78.2	0.40
80	0.020	2.61	26640	39.76	0.591	86.19	78.1	0.30
100	0.015	2.58	33300	39.16	0.588	84.92	78.4	0.24

## 4 Conclusions

In the present work, a visible transparent PMMA matrix was used as a support to embed ZnO QDs and create transparent and colorless ZnO-LSC devices, preserving the absorption and photoluminescence properties of the luminophores. The photoluminescent ZnO quantum dots were synthesized by the sol–gel method presenting average sizes of about 5.3 nm and a hexagonal wurtzite crystalline structure, as confirmed by XRD measurements. The QD emission spectrum consisted of a broad defect band extending from 425 to 750 nm, reaching a maximum at 540 nm under excitation wavelength of 350 nm. The average lifetimes of the luminescent signal increased from 2208 ns for the colloidal QDs to 2450 ns for the LSC, suggesting longer-lived excited states in the LSC due to a possible reduction of QD interaction, preventing the nonradiative recombination. An optimal ZnO-LSC-O was proposed with the combined optical properties of PMMA and colloidal ZnO QDs. A realistic solar cell model was simulated by using the software COMSOL Multiphysics to analyze the performance of the fabricated and optimized LSC-PV systems. The simulated 1.0 cm^2^ solar cell generated 14.39 mW of power under direct radiation (14.39% PCE), while both the fabricated ZnO-LSC-PV and optimized ZnO-LSC–O-PV systems were capable of generating a maximum power of 15.02 and 40.33 mW, respectively, functioning as highly transparent photovoltaic windows and employing the same photovoltaic active area. These results demonstrate that the ZnO QD-based LSC devices are capable of concentrating solar radiation and generating more power than the same solar cell directly exposed while preserving a high colorless transparency, making them promising candidates for BIPV applications.
